# Training Police Officers how to Interact effectively with those who have Autism

**DOI:** 10.1192/j.eurpsy.2025.1520

**Published:** 2025-08-26

**Authors:** V. Murphy, S. Abbott, G. Gulati

**Affiliations:** 1 Psychiatry, University College Cork, Cork, Ireland; 2 Co-Reponder Training, William James College, Boston, United States; 3 Medicine, University of Limerick, Limerick, Ireland

## Abstract

**Introduction:**

There is growing awareness of the need for Police Officers to develop skills to assist those who have Autism. The development of skills is essential for a positive outcome, be it managing an emergency call out, making an arrest or interviewing a victim of crime. This is being championed by the development of co-responder teams which consist of a Police Officer and a Mental Health Professional who are dedicated to responding to emergency calls that may have a mental health component. These teams report a high rate of call outs involving those with Autism and frequently request further training in the area.

**Objectives:**

To develop a training pilot study in Ireland which could be expanded to an international cohort of Police Officers and co-responder teams in the United States.

**Methods:**

We were invited to train a small group of Irish Police Officers. We used the opportunity to obtain feedback which we then used to identify common training needs and used to improve the presentation. This was then delivered to the Framingham Police, Boston, USA and a group of co responder trainees. Feedback was again sought and used to improve the presentation to better address the needs of the group. A presentation was delivered to a conference for those who co-ordinate co-responder teams in Law Enforcement, Universities and Colleges in the United States and feedback sought.

**Results:**

In the initial training to Irish Police Officers, feedback demonstrated improvement with significant *p*-values in all domains examined with questions e.g. ‘I understand the common difficulties those with Autism experience’ and ‘This training will help me in my day to day work’. The feedback from the conference demonstrated significant interest and engagement in the training with questions e.g. ‘The program maintained my interest’ and ‘The presenters responded to the questions and needs of the attendees’.

**Conclusions:**

There is growing recognition of the need for Police Officers to have the skills to recognise, communicate with and support those with Autism. Our program has demonstrated a need and interest of Police Officers and co-responder teams for training in this area. We have also demonstrated effectiveness of the training using feedback from the attendees.

**Disclosure of Interest:**

None Declared

